# WATER versus WATER II 2-Year Update: Comparing Aquablation Therapy for Benign Prostatic Hyperplasia in 30–80-cm^3^ and 80–150-cm^3^ Prostates

**DOI:** 10.1016/j.euros.2021.01.004

**Published:** 2021-01-31

**Authors:** David-Dan Nguyen, Neil Barber, Mo Bidair, Peter Gilling, Paul Anderson, Kevin C. Zorn, Gopal Badlani, Mitch Humphreys, Steven Kaplan, Ronald Kaufman, Alan So, Ryan Paterson, Larry Goldenberg, Dean Elterman, Mihir Desai, Jim Lingeman, Claus Roehrborn, Naeem Bhojani

**Affiliations:** a Faculty of Medicine, McGill University, Montreal, Canada; b Department of Urology, Frimley Park Hospital, Frimley, UK; c San Diego Clinical Trials, San Diego, CA, USA; d Department of Urology, Bay of Plenty District Health Board Clinical School, Tauranga, New Zealand; e Department of Urology, Royal Melbourne Hospital, Melbourne, Australia; f Division of Urology, Centre Hospitalier de l’Université de Montréal, Montreal, Canada; g Department of Urology, Wake Forest School of Medicine, Winston-Salem, NC, USA; h Department of Urology, Mayo Clinic, Phoenix, AZ, USA; i Department of Urology, Mount Sinai Hospital, New York, NY, USA; j Division of Urology, Albany Medical College, Albany, NY, USA; k Urologic Sciences, University of British Columbia, Vancouver, Canada; l Division of Urology, University of Toronto, Toronto, Canada; m USC Institute of Urology, University of Southern California, Los Angeles, CA, USA; n Institute for Kidney Stone Disease, Methodist Hospital, Indianapolis, IN, USA; o Department of Urology, UT Southwestern Medical Centre, Dallas, TX, USA

**Keywords:** Benign prostatic hyperplasia, Aquablation, Robotics

## Abstract

**Background:**

Surgical options are limited when treating large (>80 cm^3^) prostates for lower urinary tract symptoms (LUTS) due to benign prostatic hyperplasia (BPH). Open simple prostatectomy remains the most common procedure performed for large prostates. There is a need for novel surgical approaches with shorter learning curves and effective treatment. Aquablation could be this novel tool.

**Objective:**

To compare the outcome of Aquablation for 30–80-cm^3^ prostates with the outcome for 80–150-cm^3^ prostates at 2-yr follow-up.

**Design, setting, and participants:**

We used data from two trials. WATER is a prospective, double-blind, multicenter, international clinical trial comparing the safety and efficacy of Aquablation and transurethral resection of the prostate in the treatment of LUTS/BPH in men aged 45–80 yr with a prostate of 30–80 cm^3^. WATER II is a prospective, multicenter, single-arm international clinical trial of Aquablation in men with a prostate of 80–150 cm^3^.

**Intervention:**

Aquablation, an ultrasound-guided, robotically executed waterjet ablative procedure.

**Outcome measurements and statistical analysis:**

We compared 24-mo outcomes between 116 WATER and 101 WATER II study subjects. Student’s *t* test or a Wilcoxon test was used to compare continuous variables and Fisher’s test for categorical variables.

**Results and limitations:**

The International Prostate Symptom Score (IPSS) reductions at 24 mo was 14.5 points for WATER and 17.4 points for WATER II (*p* = 0.31). At baseline, the maximum urinary flow rate (Q_max_) was 9.4 and 8.7 cm^3^/s in WATER and WATER II, improving to 20.5 and 18.2 cm^3^/s, respectively (*p* = 0.60) at 24 mo. Improvements in both IPSS and Q_max_ were immediate and sustained throughout follow-up. At 2 yr, the surgical retreatment rate was 4% in WATER and 2% in WATER II.

**Conclusions:**

Aquablation is effective in patients with a prostate of 30–80 cm^3^ and patients with a prostate of 80–150 cm^3^ treated for LUTS/BPH, with comparable outcomes in both groups. It has low complication and retreatment rates at 2 yr of follow-up, with durable improvements in functional outcome.

**Patient summary:**

Outcomes of Aquablation for both small-to-moderately-sized and large prostates are similar and sustainable at 2 yr of follow-up.

## Take Home Message

Comparing data from WATER and WATER II trials, we found that Aquablation is effective in patients with a prostate of 30–80 cm^3^ and patients with a prostate of 80–150 cm^3^ treated for lower urinary tract symptoms due to benign prostatic hyperplasia, with comparable outcomes in both groups. Functional outcome improvements are durable and retreatment rates are low at 2 yr of follow-up.

## 1 Introduction

Patients with lower urinary tract symptoms (LUTS) due to benign prostatic hyperplasia (BPH) benefit from surgery if medical management fails or in specific situations such as urinary retention [1], [2]. The choice of a particular surgical modality depends on the size of the prostate. For smaller prostates, transurethral resection of the prostate (TURP) remains the historic gold standard [3] with alternative treatment options including more novel therapies such as transurethral laser photovaporization (PVP) and Aquablation [1], [2]. For prostate glands larger than 80 cm^3^, there are fewer treatment options, all hindered by non-negligible limitations. For example, open simple prostatectomy (OSP), the global gold standard for the surgical treatment of large prostates [1], [2], is associated with morbidity [4], [5]. Alternatively, laser modalities, especially holmium laser enucleation of the prostate (HoLEP), have better safety profiles than OSP for larger prostates [6], but can be time-consuming and are technically challenging, with surgeon skill influencing outcomes [7], [8], [9]. Thus, there is a gap in the existing armamentarium with regard to a surgical modality with low morbidity and reproducible outcomes independent of the surgeon.

After its approval by the US Food and Drug Administration (FDA) in 2018, Aquablation (AquaBeam System, PROCEPT BioRobotics, Redwood City, CA, USA) has shown promise to fulfill this clinical need. Aquablation is a surgeon-guided and robot-executed procedure combining multidimensional imaging, autonomous tissue removal, and a heat-free cavitating waterjet [10]. Clinical trials of Aquablation have been conducted for both small to moderately sized (30–80 cm^3^) and large (80–150 cm^3^) prostates, and there have also been reports of real-world experience with this approach [11], [12]. Previous subgroup and pooled analyses of clinical trials by our group demonstrated that the short-term effectiveness of Aquablation is independent of prostate size and independent of intraoperative surgeon skill [13], [14].

The aim of the present study was to update the findings from the previous pooled analysis to determine if the effectiveness of Aquablation is independent of prostate size and persists with durability at 2 yr of follow-up [14]. To this end, we compared data from two separate clinical trials, one studying Aquablation for enlarged prostates between 30 and 80 cm^3^ and the other studying the procedure for prostates between 80 and 150 cm^3^.

## 2 Patients and methods

### 2.1 Clinical trials and Aquablation intervention

WATER (Waterjet Ablation Therapy for Endoscopic Resection of Prostate Tissue; NCT02505919) is a prospective, double-blind, multicenter, international clinical trial comparing the safety and efficacy of Aquablation to TURP for the treatment of LUTS due to BPH in men aged 45–80 yr with a prostate volume between 30 and 80 cm^3^ as measured via transrectal ultrasound [15]. Participants were enrolled at 17 centers between November 2015 and December 2016. Eligible study participants had moderate to severe LUTS, defined as an International Prostate Symptom Score (IPSS) of ≥12 and maximum urinary flow rate (Q_max_) of ≤15 ml/s. Participants were excluded from analysis if they had a body mass index ≥42 kg/m^2^; a history of prostate or bladder cancer, neurogenic bladder, bladder calculus, or clinically significant bladder diverticulum; active infection; treatment for chronic prostatitis; diagnosis of urethral stricture, meatal stenosis, or bladder neck contracture; a damaged external urinary sphincter; stress urinary incontinence; postvoid residual volume (PVR) >300 ml or urinary retention; self-catheterization use; and/or prior prostate surgery. Anticoagulant or bladder anticholinergic users and participants with severe cardiovascular disease were also excluded.

WATER II (NCT03123250) is a prospective, multicenter, international clinical trial of Aquablation for the surgical treatment of LUTS/BPH in men aged 45–80 yr with a prostate volume between 80 and 150 cm^3^ as measured via transrectal ultrasound [16]. Patients using catheters and those who had prior surgery were allowed to participate in WATER II. All other inclusion and exclusion criteria were the same as in WATER. Both trials are currently under active follow-up. Participants were enrolled at 13 US and three Canadian sites between September 2017 and December 2017. Patients on catheter use and those who had prior surgery were allowed to participate in WATER II, unlike WATER. All other inclusion and exclusion criteria were the same as in WATER.

**Fig. 1:**
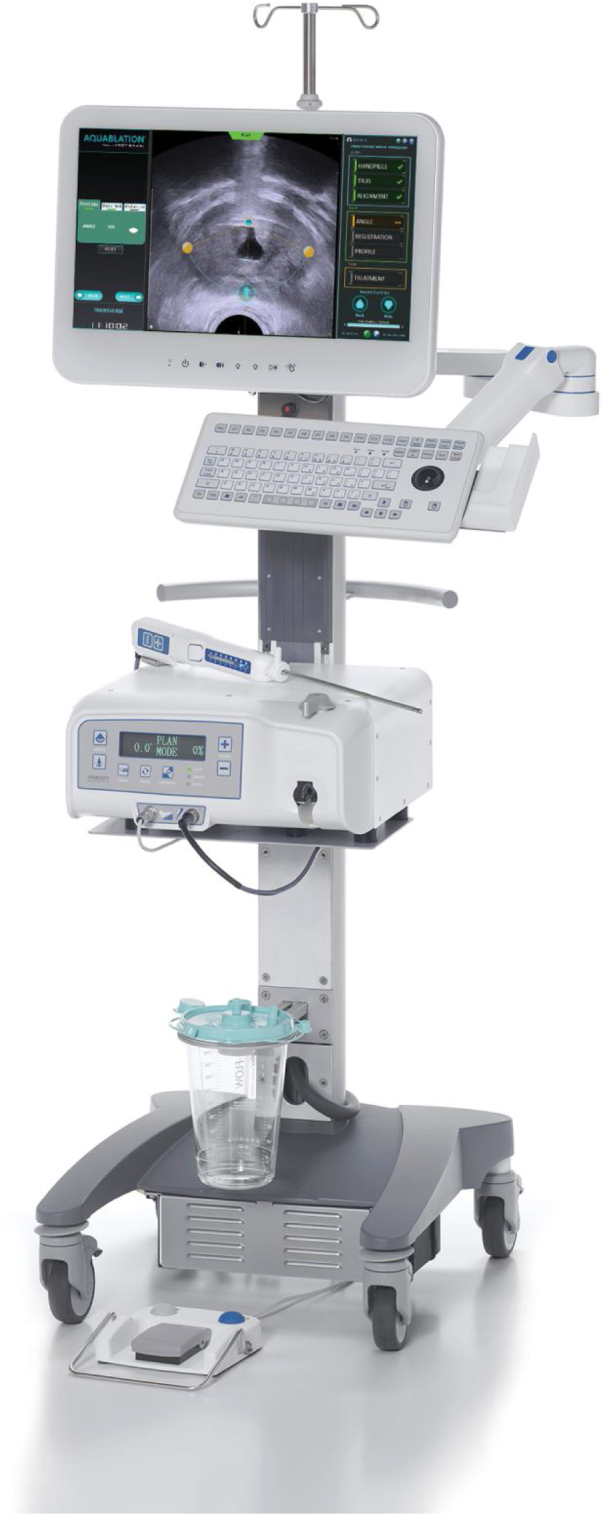
The Aquabeam system.

The Aquablation procedure was performed using the AquaBeam System as previously described [10]. Figure 1 shows the AquaBeam device.

### 2.2 Study parameters

At baseline, the IPSS and Incontinence Severity Index (ISI) questionnaires were completed by trial participants. Uroflowmetry, PVR measurements, and standard laboratory blood assessment were also undertaken. These questionnaires and measurements were repeated at scheduled follow-up visits at 1, 3, 6, 12, and 24 mo. Prostate-specific antigen (PSA) was assessed at baseline and 6 mo and then annually. Other questionnaires not repeated up to 24 mo were not included in this analysis. Adverse events occurring up to 12 mo after the initial treatment were adjudicated for severity by a clinical events committee. Events were assigned a Clavien-Dindo grade.

### 2.3 Statistical analysis

Baseline characteristics for each trial were compared using a Student *t* test and Wilcoxon signed-rank test for normally and non-normally distributed continuous variables, respectively. Fisher’s test was used for categorical variables. Repeated-measures analysis of variance was used to compare longitudinal responses at different time points, adjusting for patient clustering.

All statistical analyses were performed using the R programming language (R Foundation for Statistical Computing, Vienna, Austria). The level of significance was set at a two-sided *p* = 0.05. Analyses through month 24 are reported here.

## 3 Results

### 3.1 Baseline demographics

At 2 yr, 117 WATER and 101 WATER II patients were available for analysis. Baseline characteristics for participants in both clinical trials were similar with the exception of prostate volume and PSA, which were greater in the WATER II study (both *p* < 0.001). Baseline demographic data are presented in Table 1.

**Table 1:** Baseline characteristics by trial

Characteristic	WATER (*n* = 117)	WATER II (*n* = 101)	*p* value
Mean age, yr (SD)	65.9 (7.3)	67.5 (6.6)	0.0854
Mean body mass index, kg/m^2^ (SD)	28.4 (4.1)	28.3 (4.1)	0.8231
Mean prostate-specific antigen, g/dl (SD)	3.7 (3)	7.1 (5.9)	<0.0001
Mean prostate size, cm^3^ (SD) [range]	54.1 (16.3) [25–80]	107.4 (20.2) [80–150]	<0.0001
Median lobe present, *n* (%)	58 (50)	73 (72)	0.0044
Intravesical component	42/58 (72)	69/73 (95)	
Use of catheter within 45 d before consent, *n* (%)	– a	16 (16)	–
Mean IPSS (SD)	22.9 (6)	23.2 (6.3)	0.6933
Mean IPSS QOL (SD)	4.8 (1.1)	4.6 (1)	0.1805

IPSS = International Prostate Symptom Score; QOL = quality of life domain; SD = standard deviation.

a Patients reporting urinary catheter use in the 14 d before evaluation or with history of intermittent self-catheterization were excluded from WATER.

### 3.2 Perioperative outcomes

Perioperative outcomes were previously extensively analyzed in the 1-yr comparison paper [14]. The mean procedure time was 32.8 min (standard deviation [SD] 16.5 min; range 10–96 min) in WATER and 37.4 min (SD 13.5 min; range 15–97 min) in WATER II (*p* = 0.027). The mean length of stay was 1.4 d for the WATER group and 1.6 d for the WATER II group (*p* = 0.007). The mean catheter time was 2 d (SD 2.3 d; range 0.25–19 d) in WATER and 3.9 d (SD 3.6 d; range 0.7–30 d) in WATER II (*p* < 0.001).

### 3.3 Functional outcomes

**Fig. 2:**
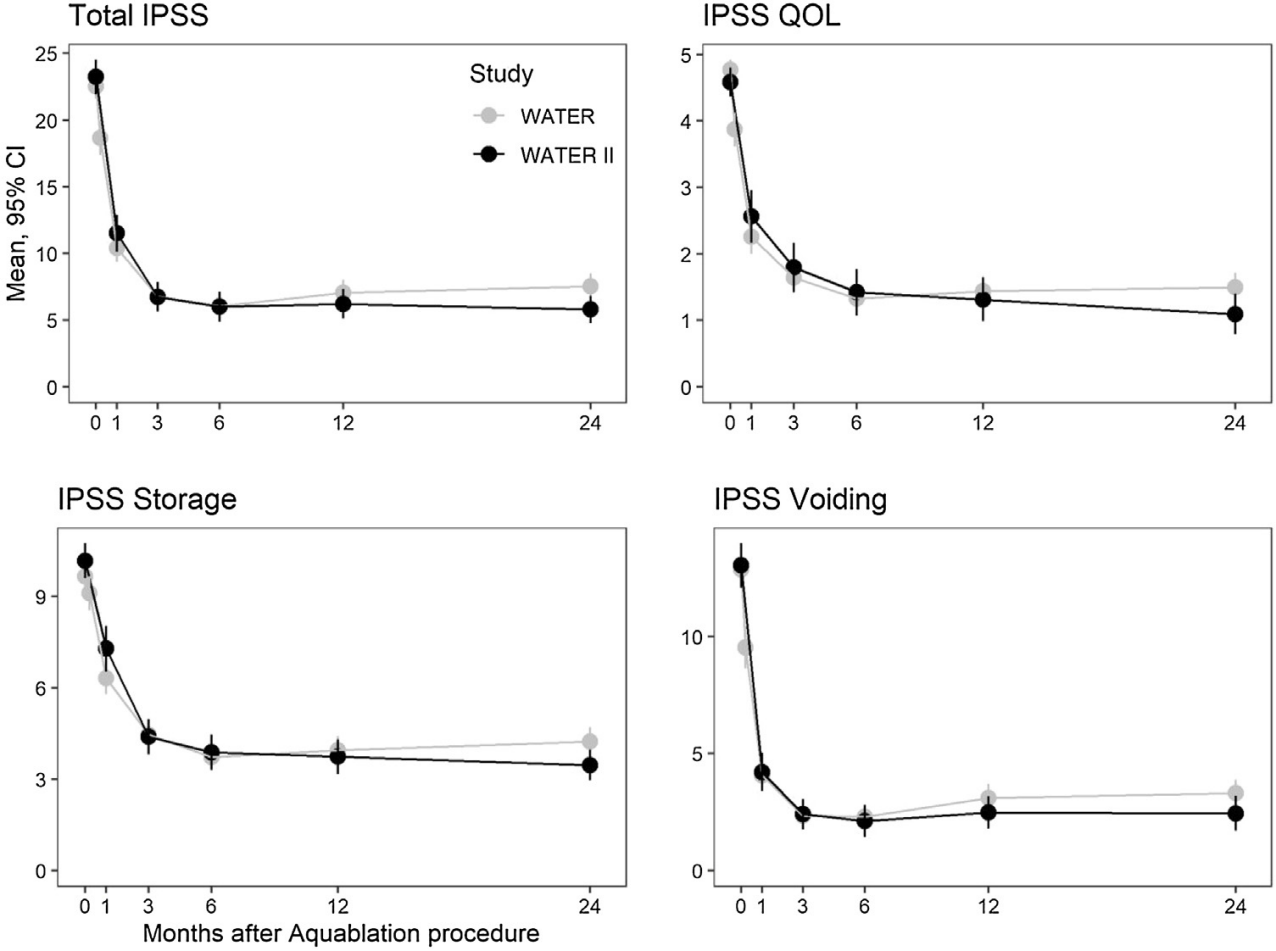
International Prostate Symptom Score (IPSS), IPSS quality of life (QOL), and IPSS storage and voiding subscale scores by month after Aquablation in WATER and WATER II. CI = confidence interval.

Mean IPSS scores improved in WATER and WATER II from 22.9 and 23.2 at baseline to 8.4 and 5.8 at 24 mo, respectively. The corresponding mean 24-mo improvements were 14.7 (95% confidence interval [CI] 13.3–16) and 17.4 (95% CI 15.7–19.1) points; both changes were highly statistically significant (*p* < 0.0001). The mean IPSS quality of life (QOL) score improved from 4.8 and 4.6 points at baseline to 1.6 and 1.1 points at 24 mo (improvements of 3.4 and 3.3 points, respectively; both *p* < 0.0001). Mean IPSS storage and voiding subdomain scores also improved significantly. IPSS scores are presented in Figure 2.

**Fig. 3:**
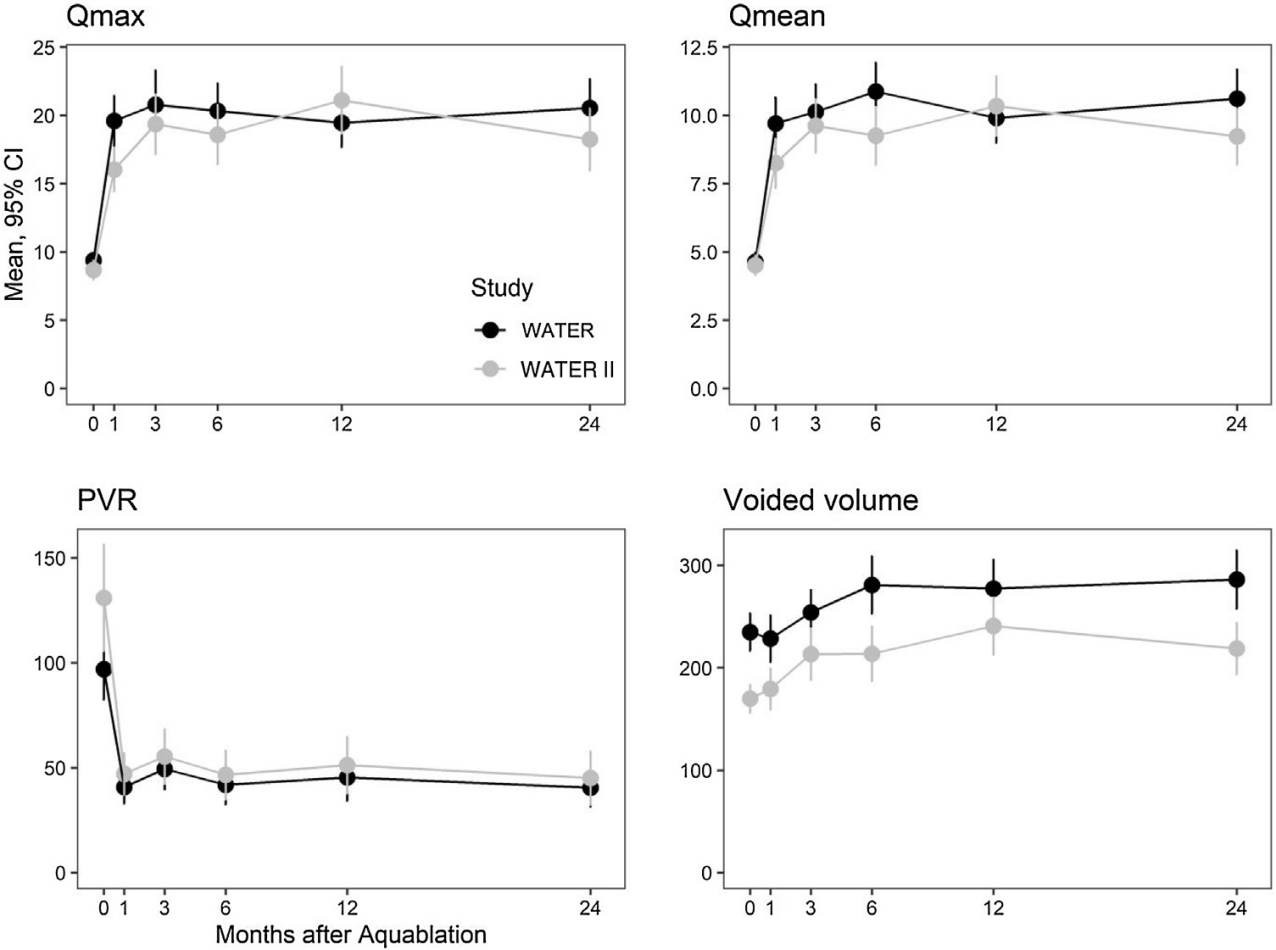
Uroflowmetry parameters by month after Aquablation in WATER and WATER II. PVR = postvoid residual volume. CI = confidence interval.

Uroflowmetry measures also showed improvement. Mean Q_max_ improved from 9.4 and 8.7 cm^3^/s at baseline in WATER and WATER II to 20.5 and 18.2 cm^3^/s at 24 mo, representing improvements of 11.2 and 9.7 cm^3^/s, respectively (*p* < 0.0001). Mean PVR decreased from 97 and 131 cm^3^ to 40 and 45 cm^3^ at 24 mo (decrease of 57 and 96 cm^3^; *p* < 0.0001), respectively. Uroflowmetry results are presented in Figure 3.

Repeated-measures analysis of variance for score changes between months 1 and 24 showed no statistically significant differences between the studies in the following measures: IPSS (*p* = 0.31), IPSS QOL (*p* = 0.30), IPSS storage (*p* = 0.22) and voiding (*p* = 0.49) subscales, Q_max_ (*p* = 0.60), Q_mean_ (*p* = 0.26), and voided volume (*p* = 0.40). The improvement in PVR was greater in WATER II than in WATER (*p* = 0.02).

At 2 yr, 2.6% of the WATER patients had a stenosis of the bladder neck and 0.9% had a stenosis of the urethra. At 2 yr, 0% of WATER patients had a stenosis of the bladder neck and 2.0% had a stenosis of the urethra.

### 3.4 Retreatment rates and PSA changes

**Fig. 4:**
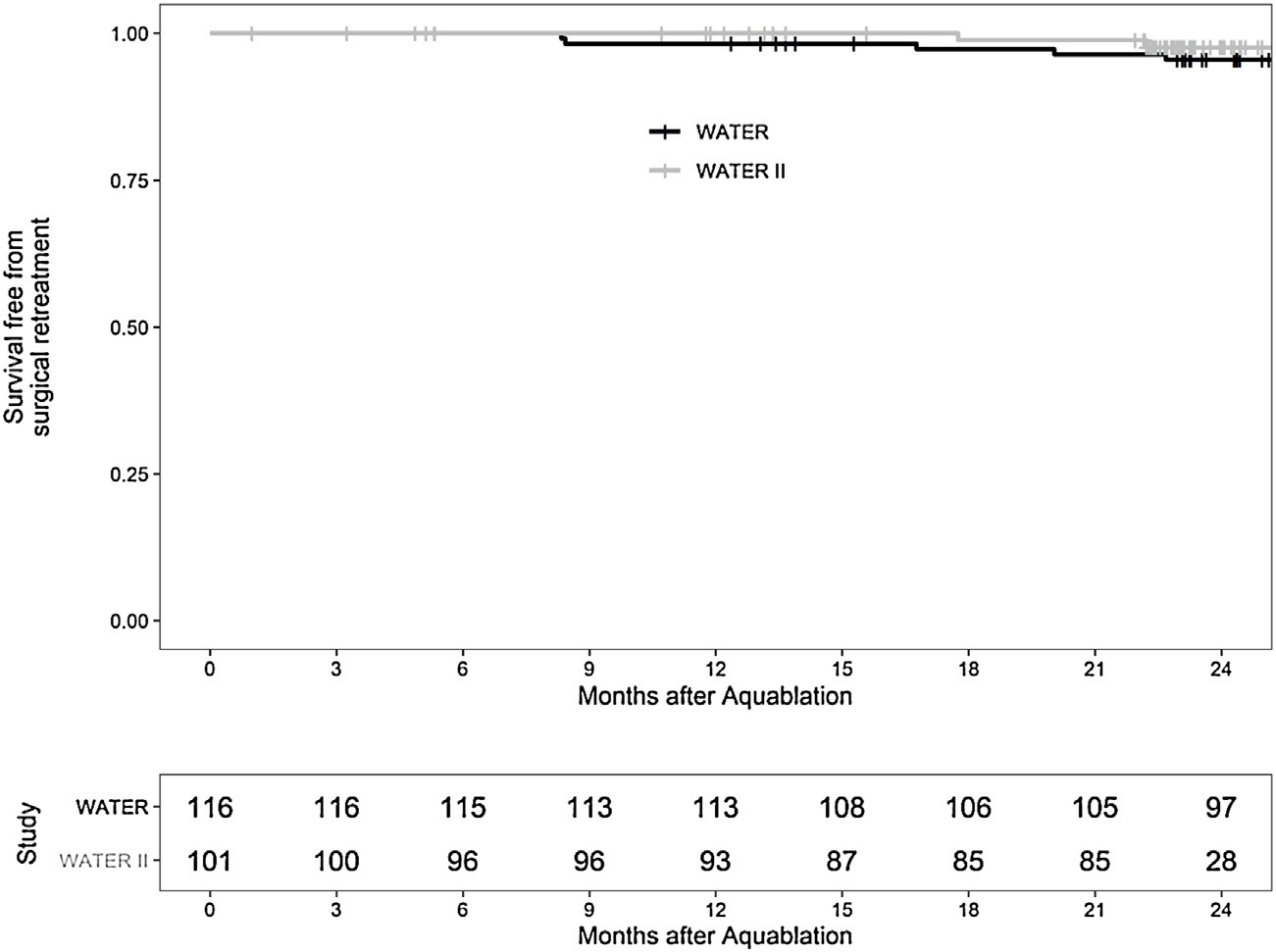
Retreatment-free time for symptomatic benign prostatic hyperplasia in WATER and WATER II.

**Fig. 5:**
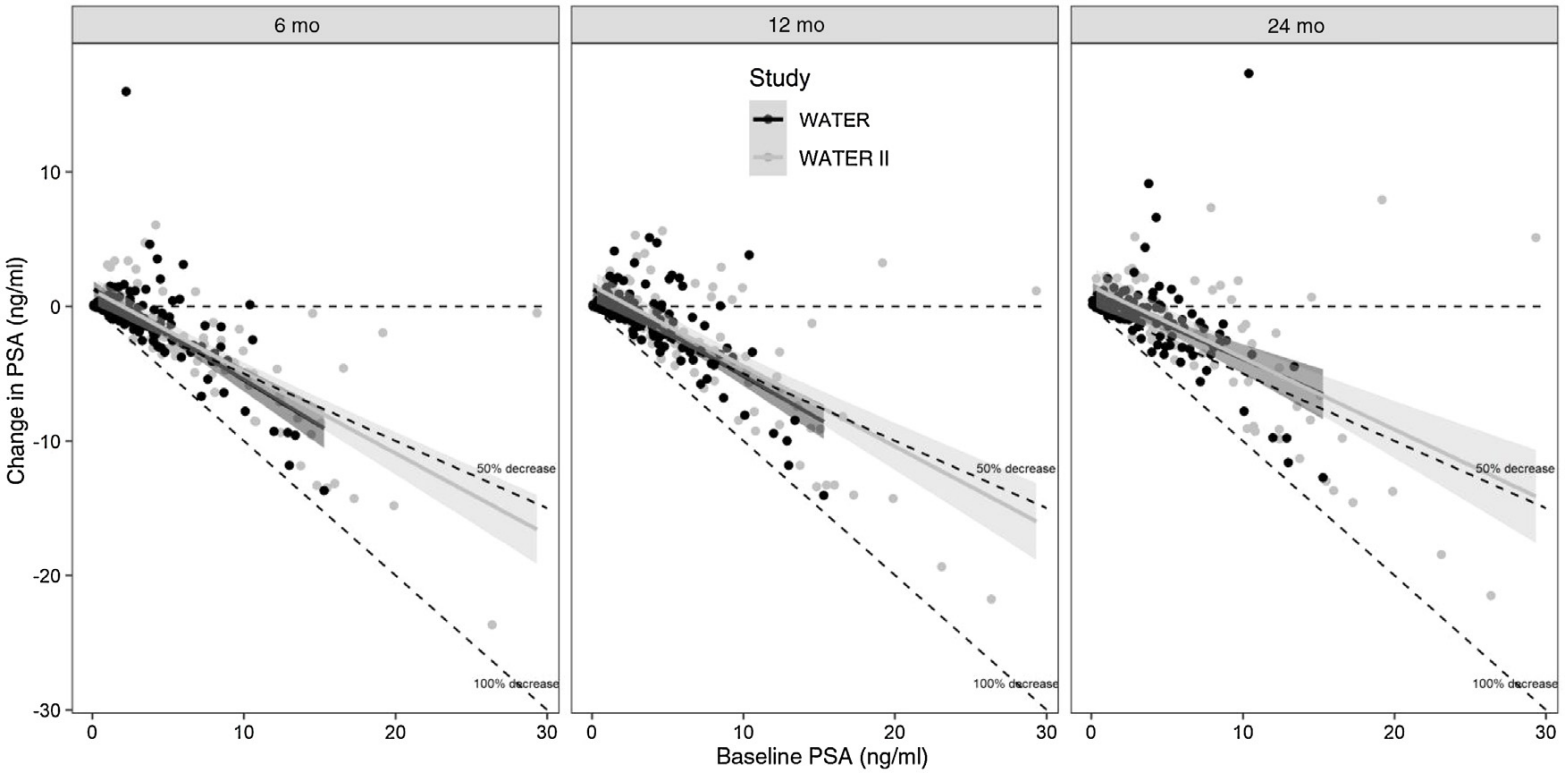
Change in prostate-specific antigen (PSA) at 6, 12, and 24 mo. *The regression line is at or below the 50% reduction line for all time points.

At 2 yr, the Kaplan-Meier freedom from surgical retreatment was 95.7% in WATER and 98.0% in WATER II, with five and two patients, respectively, requiring surgical retreatment. Medical BPH retreatment (defined as initiation of an α blocker or 5-α reductase inhibitor after surgery) at 2 yr occurred in 4.3% (*n* = 5) of patients in WATER and 5.9% (*n* = 6) in WATER II. Figure 4 shows the Kaplan-Meier surgical retreatment–free survival curve. Regarding changes in PSA, baseline PSA was 3.7 ng/ml in WATER and 7.1 ng/ml in WATER II; at 2 yr, PSA was 3.0 ng/ml in WATER and 4.9 ng/ml in WATER II. Figure 5 presents the change in PSA at 6, 12, and 24 mo; the regression line is at or below the 50% reduction line for all time points.

## 4 Discussion

With the large spectrum of prostate volumes and configurations, coupled with inconsistent uptake and surgical expertise for the various modalities available, there is a need for a surgical modality with volume-independent effectiveness, durable, and reproducible outcomes independent of the surgeon, and less morbidity when treating prostates larger than 80 cm^3^. Our updated pooled analysis of 2-yr Aquablation trial data suggests that the clinical benefits of Aquablation for LUTS due to BPH in small to moderately sized prostates (30–80 cm^3^) transfer to large prostates (80–150 cm^3^) and are sustainable up to 2 yr, with a very low retreatment rate. Achieving these outcomes does not require significant surgeon experience, regardless of prostate size, considering that nine out of 16 WATER II sites had never performed an Aquablation procedure before the start of the trial [14].

At baseline, there were no statistically significant differences in characteristics between the two cohorts other than factors related to prostate size such as PSA. There were no clinically relevant differences in terms of procedural outcomes. The time from ultrasound probe insertion to insertion of the catheter and the resection time were longer for larger prostates, but only by 15 and 4 min, respectively. This increase in operative time with prostate size is much smaller relative to other surgical modalities owing to the robot-controlled efficiency and precision of the planning [9]. There were similar trends for IPSS, IPSS QOL, and Q_max_ results between the two trials. PVR changes, while also trending similarly, were statistically greater in WATER II, probably because baseline values were substantially higher, indicating a higher likelihood of retention related to bladder outlet obstruction from larger prostates.

In this updated pooled analysis of Aquablation trials, retreatment rates remained low, demonstrating the durability of Aquablation outcomes at 2 yr for prostates of 30–80 cm^3^ and 80–150 cm^3^. Only 9% of patients in WATER and 8% in WATER II required a secondary surgical procedure or medical treatment. These retreatment rates are similar to or lower than those reported for GreenLight PVP [17], [18], prostatic urethral lift [19], [20], and convective radiofrequency thermal therapy [21], but are slightly higher than the surgical retreatment rates reported for HoLEP and TURP [6], [22]. Thus, Aquablation demonstrates acceptable durability for prostate sizes of both 30–80 cm^3^ and 80–150 cm^3^ at 2 yr.

Over the past decade, HoLEP has remained widely regarded as the only volume-independent surgical treatment option for bladder outlet obstruction [23]. However, its universal adoption has been hindered by its steep learning curve and the need for fellowship training, among other factors [24], [25]. While the number of HoLEP cases needed to reach a steady state (plateau) varies according to a number of factors such as previous surgical experience, it has generally been reported that the HoLEP learning curve is between at least 20 and 30 cases [24], [25], [26]. Endoscopic enucleation approaches with other lasers similarly require approximately 20–40 cases for the learning curve [27]. While the success of HoLEP relies on the surgeon’s skill, Aquablation only relies on the surgeon’s decision-making ability as the procedure is surgeon-guided, automated, and robotically executed, and provides live ultrasound imaging throughout the procedure. This potentially minimizes surgeon-to-surgeon variability [28]. In addition, it is important to mention that experience with Aquablation in the WATER and WATER II trials was limited. For example, 14 out of 17 centers and nine out of 16 centers had no prior experience in the WATER and WATER II trials, respectively. However, it is important to note that PSA reduction is greater with HoLEP, probably because HoLEP provides more efficient ablation [23].

However, while Aquablation may be more accessible technically, it has its own challenges with regard to uptake, as reimbursement for the procedure has been lacking in the USA. Aquablation was only recently covered by Medicare, nearly 3 yr after FDA approval [29]. The Canadian Agency for Drugs and Technologies in Health, an independent, not-for-profit organization created by the Canadian government that provides health care decision-makers with objective evidence on the use of health technologies, has suggested that while there may be benefits to Aquablation, real-world evidence confirming these potential benefits and long-term cost-effectiveness analyses are still needed [30]. Thus, in the absence of widespread reimbursement and coverage of the procedure and of stronger evidence of its cost-effectiveness to convince health care systems to cover it, Aquablation is currently limited to certain settings where other sources of funding (such as private philanthropy) or patients cover the costs of the procedure. Beyond access to the technology, other limitations of Aquablation include the absence of pathological anatomy samples.

Our analysis of the Aquablation trial data is not without limitations. First, as WATER II was a single-arm study, it was not compared to another surgical modality. While this is important for comparative effectiveness, this was not the intent of our analysis, which compared Aquablation between small to moderately sized prostates and larger prostates. Second, while we demonstrate the durability of our previous findings at 2-yr follow-up, longer-term data from these trials are still needed to demonstrate the volume-independent durability of the treatment outcomes.

## 5 Conclusions

Aquablation therapy clinically normalizes outcomes among patients regardless of prostate size or shape. The advantages of Aquablation, namely short operative times and smooth learning curves for clinical outcomes, are comparable for both small-to-moderately-sized and large prostates. These findings suggest that the effectiveness of Aquablation is independent of prostate size and that outcomes are durable for up to 2 yr of follow-up.

  ***Author contributions***: Naeem Bhojani had full access to all the data in the study and takes responsibility for the integrity of the data and the accuracy of the data analysis.

  *Study concept and design*: Nguyen, Bhojani.

*Acquisition of data*: All authors.

*Analysis and interpretation of data*: All authors.

*Drafting of the manuscript*: Nguyen, Bhojani.

*Critical revision of the manuscript for important intellectual content*: All authors.

*Statistical analysis*: Nguyen, Bhojani.

*Obtaining funding*: All authors.

*Administrative, technical, or material support*: Nguyen, Bhojani.

*Supervision*: Barber, Bidair, Gilling, Anderson, Zorn, Badlani, Humphreys, Kaplan, Kaufman, So, Paterson, Goldenberg, Elterman, Desai, Lingeman, Roehrborn, Bhojani.

*Other*: None.

  ***Financial disclosures:*** Naeem Bhojani certifies that all conflicts of interest, including specific financial interests and relationships and affiliations relevant to the subject matter or materials discussed in the manuscript (eg, employment/affiliation, grants or funding, consultancies, honoraria, stock ownership or options, expert testimony, royalties, or patents filed, received, or pending), are the following: Mihir Desai and Mo Bidair are consultants for PROCEPT BioRobotics. Kevin Zorn and Naeem Bhojani have been paid for a training session at AUA 2018. The remaining authors have nothing to disclose.

  ***Funding/Support and role of the sponsor*:** The WATER and WATER II clinical trials are funded by PROCEPT BioRobotics.

## CRediT authorship contribution statement

**David-Dan Nguyen:** Conceptualization, Methodology, Formal analysis, Writing - original draft, Visualization. **Neil Barber:** Investigation, Writing - review & editing. **Mo Bidair:** Investigation, Writing - review & editing. **Peter Gilling:** Investigation, Writing - review & editing. **Paul Anderson:** Investigation, Writing - review & editing. **Kevin C. Zorn:** Investigation, Writing - review & editing. **Gopal Badlani:** Investigation, Writing - review & editing. **Mitch Humphreys:** Investigation, Writing - review & editing. **Steven Kaplan:** Investigation, Writing - review & editing. **Ronald Kaufman:** Investigation, Writing - review & editing. **Alan So:** Investigation, Writing - review & editing. **Ryan Paterson:** Investigation, Writing - review & editing. **Larry Goldenberg:** Investigation, Writing - review & editing. **Dean Elterman:** Investigation, Writing - review & editing. **Mihir Desai:** Investigation, Writing - review & editing. **Jim Lingeman:** Investigation, Writing - review & editing. **Claus Roehrborn:** Investigation, Writing - review & editing. **Naeem Bhojani:** Conceptualization, Methodology, Investigation, Formal analysis, Writing - review & editing, Supervision.
